# Towards Optimal Platform-Based Robot Design for Ankle Rehabilitation: The State of the Art and Future Prospects

**DOI:** 10.1155/2018/1534247

**Published:** 2018-03-15

**Authors:** Qing Miao, Mingming Zhang, Congzhe Wang, Hongsheng Li

**Affiliations:** ^1^School of Mechanical and Electrical Engineering, Wuhan University of Technology, Wuhan, China; ^2^State Key Lab of Digital Manufacturing Equipment and Technology, Huazhong University of Science and Technology, Wuhan, China; ^3^Department of Mechanical Engineering, The University of Auckland, Auckland, New Zealand; ^4^School of Advanced Manufacturing Engineering, Chongqing University of Posts and Telecommunications, Chongqing, China

## Abstract

This review aims to compare existing robot-assisted ankle rehabilitation techniques in terms of robot design. Included studies mainly consist of selected papers in two published reviews involving a variety of robot-assisted ankle rehabilitation techniques. A free search was also made in Google Scholar and Scopus by using keywords “ankle^∗^,” and “robot^∗^,” and (“rehabilitat^∗^” or “treat^∗^”). The search is limited to English-language articles published between January 1980 and September 2016. Results show that existing robot-assisted ankle rehabilitation techniques can be classified into wearable exoskeleton and platform-based devices. Platform-based devices are mostly developed for the treatment of a variety of ankle musculoskeletal and neurological injuries, while wearable ones focus more on ankle-related gait training. In terms of robot design, comparative analysis indicates that an ideal ankle rehabilitation robot should have aligned rotation center as the ankle joint, appropriate workspace, and actuation torque, no matter how many degrees of freedom (DOFs) it has. Single-DOF ankle robots are mostly developed for specific applications, while multi-DOF devices are more suitable for comprehensive ankle rehabilitation exercises. Other factors including posture adjustability and sensing functions should also be considered to promote related clinical applications. An ankle rehabilitation robot with reconfigurability to maximize its functions will be a new research point towards optimal design, especially on parallel mechanisms.

## 1. Introduction

Conventional rehabilitation programs for musculoskeletal and neurological disabilities require cooperative and intensive efforts from both therapists and patients [1, 2]. This emphasises the need for novel rehabilitation techniques to enable therapists to provide efficacious treatment interventions without increasing the burden on staff and resources. Robotics technology can provide an overdue transformation of rehabilitation clinics from labor-intensive operations to technology-assisted ones, as well as a rich stream of data that can facilitate patient diagnosis, customization of therapies, and maintenance of patient records [1].

Saglia et al. [3] have summarized the rehabilitation protocol for ankle injuries. In the early stage of ankle therapy, the patient can hardly move his/her foot, and thus, a passive exercise is mostly needed, where the trajectory parameters, such as speed, amplitudes, and number of repetitions, can be set by the physiotherapist. Active exercises can be involved next to help the patient to fully regain his/her ankle range of motion (ROM). Strength training includes both isometric and isotonic exercises. The last stage of rehabilitation requires the patient to conduct proprioceptive training, such as the balance exercises.

A systematic review by Zhang et al. [4] has demonstrated that most robot-assisted ankle rehabilitation techniques are effective in improving ankle performance or gait function after a period of training therapy. Existing ankle rehabilitation robots mainly include wearable devices and platform-based ones. Wearable devices typically take the form of a robotic orthosis [5] or an exoskeleton [6], for correcting the gait pattern of patients. Platform-based devices have stationary bases and can be designed with a single range of motion (DOF) [7] or multiple DOFs [3, 8].

Zhang et al. [4] also concluded that the most effective robot-assisted intervention for ankle rehabilitation is vague due to the lack of universal evaluation criteria. However, the potential of existing ankle rehabilitation robots can be compared and analyzed in terms of structure design by considering the actual ankle anatomy and motion. The purpose of this review is to provide a comprehensive investigation on the structural designs of existing ankle rehabilitation robots, thus promoting the development of advanced robotic system for ankle therapy. To the best of the authors' knowledge, this is the first ever attempt, wherein a comprehensive comparison in the field of ankle rehabilitation robots in terms of robot design is made. This review is organized as follows. It starts with the search strategy used for literatures, followed by descriptive results of a variety of robot designs. The Discussion compares existing robot-assisted ankle rehabilitation techniques in terms of robot design, introduces optimal robot designs for certain applications, and describes the limitations of this review. Lastly, the Conclusion of this review summarizes.

## 2. Search Strategy

Included studies mainly come from the selected papers in a systematic review [4] that focuses on a variety of ankle rehabilitation robots. These devices can follow predefined trajectories for range of motion and strength exercises. The gait training devices involving the ankle, reviewed by Cao et al. [9], are also included. To ensure all typical ankle rehabilitation devices are covered, an additional search was made in Google Scholar and Scopus by using keywords “ankle^∗^,” “robot^∗^,” “rehabilitat^∗^,” or “treat^∗^.” The search is limited to English-language articles published between January 1980 and September 2016. Valuable references listed in relevant publications were further screened. Since this review focuses on comparative analysis of existing ankle rehabilitation devices in terms of robot design, only one of the studies with the same robot-assisted ankle rehabilitation technique will be included and discussed. The inclusion criterion is that if the study clearly presented the robot design, and the most recent one will be selected when there are multiple studies meeting the inclusion criterion. Included prototypes are either intelligently controlled or have multiple DOFs and have also been experimentally validated. Ankle devices specially designed for assessment [10, 11] will not be included in this review, as well as traditional powered ankle-foot orthoses [12–14]. It should be noted here that conventional powered ankle-foot orthoses mostly used springs to provide adaptive power to the joint for motion control and torque assistance.

## 3. Results

**Figure 1:**
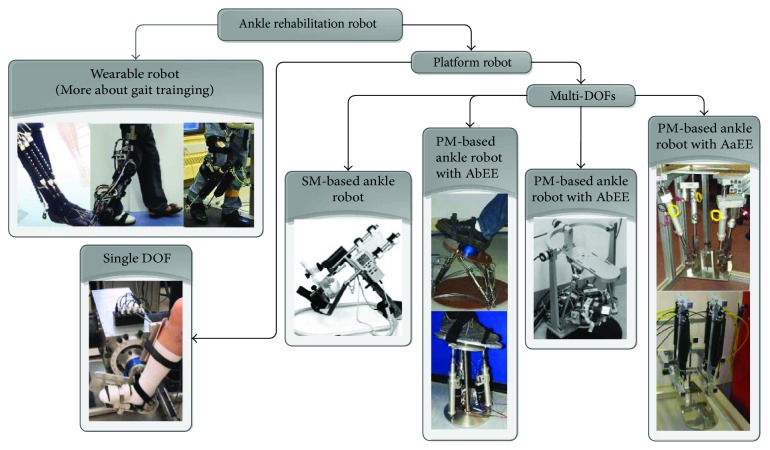
Classification chart of existing rehabilitation robots developed for ankle therapy (PM: parallel mechanism; SM: serial mechanism; AbEE: actuators below the end effector; AaEE actuators above the end effector). Pictures adopted from studies [5–8, 15–20] with permission.

Figure 1 presents typical types of robotic systems developed for ankle rehabilitation. Existing ankle rehabilitation robots are mainly classified into two categories based on the mobility of the device during operation. They are wearable robots and platform-based ones. The MIT Anklebot [6], the bio-inspired soft ankle robotic device [5], and the knee-ankle exoskeleton by Yu et al. [15] belong to the wearable robots group. These wearable ankle exoskeletons are mostly developed to correct the user's gait pattern. The other group consists of platform-based robots that usually have a fixed platform and a moving footplate with one or multiple DOFs [7, 8, 16–19]. A single-DOF device is usually actuated by a rotating motor for a specific application; for example, the device developed by Zhang et al. [7] for ankle stretching, while a multiple DOF robot is mostly based on a parallel mechanism (PM) actuated by linear actuators [8, 16–18] or motor-based linkages [19]. The OptiFlex Ankle Continuous Passive Motion (CPM) [20] has two DOFs using a serial mechanism (SM), as an exception. Further, already-developed parallel ankle rehabilitation robots fall into two categories based on their rotation centers. One kind of parallel ankle platforms have their actuators below the end effector (AbEE). The others are driven with actuators above the end effector (AaEE). A list of studies with typical ankle robot designs is provided in Table 1, and comparative analysis will be detailed in the next section in terms of their advantages and disadvantages.

## 4. Discussion

### 4.1. Comparison Analysis on Existing Robotic Designs for Ankle Rehabilitation

Wearable ankle rehabilitation robotic systems usually have one to three DOFs [5, 6, 15]. Yu et al. [15] developed a knee-ankle-foot robot for gait training of stroke patients. Park et al. [5] designed a bio-inspired soft wearable robotic device for assisting dorsiflexion and plantarflexion as well as inversion and eversion to provide assistance during gait. The main feature of the device is the use of soft structure for providing assistance without restricting natural ankle motion. The MIT Anklebot by Roy et al. [6] was actively actuated in two of the robot's three DOFs (dorsiflexion/plantarflexion and inversion/eversion) for correcting the gait pattern. These wearable robotic systems are preferably used in a mobile way or combined in a lower limb exoskeleton system. By contrast, platform-based robotic systems focus solely on ankle exercises rather than the gait training.

A variety of platform-based robotic systems have been proposed and developed based on the consideration of ankle anatomy. The simplest representation of ankle motion is a hinge joint in the sagittal plane. A platform following this model is the ankle stretching device developed by Zhang et al. [7]. This is reasonable for a specific application such as muscle stretching along dorsiflexion, although the hinge model ignores ankle inversion and eversion. The biaxial model [24] considers foot motion to be equivalent to rotations about two hinges in series, which is similar to the OptiFlex Ankle CPM system reviewed in [20]. While this device is able to provide anatomical motion for the ankle to enhance patient comfort and compliance, its workspace cannot cover the actual foot motion since the axes of rotation are skewed and angular displacements in the ankle and subtalar joints produce rotations of the foot in all three planes. Lundberg et al. [25] found that the axes of ankle joint vary with the foot orientation, which may also impede the application of the biaxial mode. The ankle robot developed by Saglia et al. [3] has the same DOFs (dorsiflexion/plantarflexion and inversion/eversion) as the OptiFlex Ankle CPM system. One difference is that its axes of rotation can be adjusted by advanced adaptive control techniques. With regard to the kinematics of the ankle-foot complex, Siegler et al. [26] demonstrated that neither the ankle joint nor the subtalar joint was acting as an ideal hinge joint with fixed axes of rotation. The motion of the ankle-foot complex is actually the result of rotations about both joints. The contribution of the ankle joint to dorsiflexion/plantarflexion is larger than that of the subtalar joint, the contribution of the subtalar joint to inversion/eversion is larger than that of the ankle joint, and the ankle and subtalar joints have approximately equal contributions to adduction/abduction. From this point, a three-DOF rehabilitation system is more suitable for comprehensive ankle therapy.

**Figure 2:**
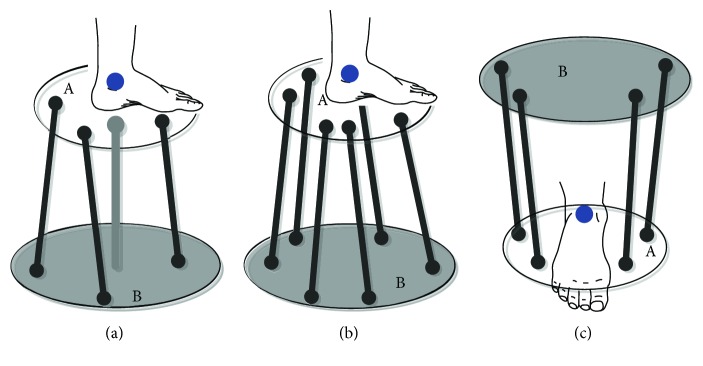
Typical parallel ankle robot designs with or without aligned rotation center between the robot and the ankle joint: (a) misaligned rotation center, (b) can be programed to have aligned rotation center but will sacrifice workspace, and (c) aligned rotation center without sacrificing workspace. The blue dots represent the ankle joint. A: the moving platform; B: the fixed platform.

Saglia et al. [3] have developed advanced control techniques to perform patient-active exercises with and without motion assistance. However, a major concern with this kind of device is the misaligned rotation center between the robot and the ankle joint, as shown in Figure 2(a). This robot is redundantly actuated by three linear actuators with a passive central strut (the grey bar in the center). Its rotation center is located at the top of the strut, while the ankle joint is obviously above the end effector. This can cause difficulties in defining the training protocol and even injuries to the human users. While the Rutgers Ankle developed by Girone et al. [18] can be programed to have an aligned rotation center as the ankle joint, such a configuration suffers from a limited workspace unless using a bulky mechanism, as shown in Figure 2(b). These two mechanisms have the design of AbEE. To distinguish the robot design (AbEE), the mechanism with its actuators above the end effector is named AaEE. This design is derived by mimicking the biological musculoskeletal system of the human foot and lower leg. A typical example is the parallel ankle rehabilitation robot developed by Tsoi et al. [16], as shown in Figure 2(c). In this design, the rotation center of the robot can be easily controlled to be aligned with the ankle joint, which means that the patients can put their shanks on a leg holder during the training and discomfort will not be brought to the human users.

**Figure 3:**
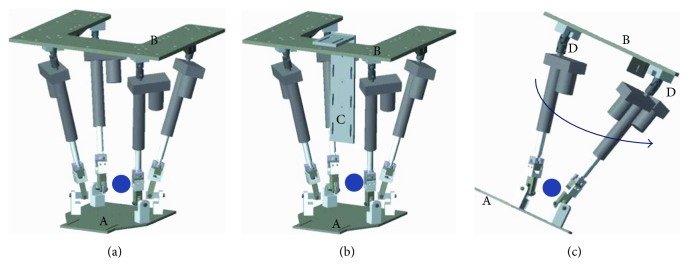
The parallel ankle robot developed by Tsoi et al. [16]: (a) the robot with six DOFs without the leg holder, (b) the robot with three rotational DOFs with the shank attached to the leg holder, and (c) the robot in a tilted position. The blue arrow line refers to the slide direction due to gravity; the blue dots represent the ankle joint. A: the moving platform; B: the fixed platform; C: leg holder; D: spherical joint.

**Figure 4:**
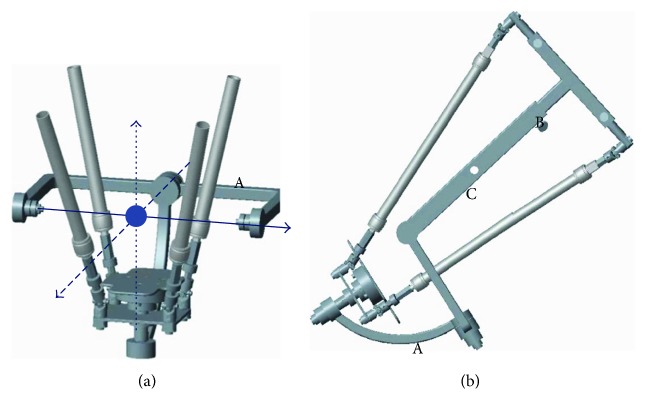
The parallel ankle robot developed by Jamwal et al. [8] and Zhang [21]: (A) the moving platform is actually a three-link serial mechanism with three rotational DOFs, (B) the fixed platform, and (C) the rotation axis of the robot structure for different postures. The blue dot represents the ankle joint; the blue solid line is the rotation axis of ankle dorsiflexion and plantarflexion; the blue dashdot line is the rotation axis of ankle inversion and eversion; the blue dot line is the rotation axis of ankle adduction and abduction.

For an ankle rehabilitation robotic system with three rotational DOFs, the parallel mechanism is more often employed than serial mechanisms due to some features (safe workspace and large torque generation capacity). The multi-DOF ankle robot with AaEE has been considered to be suitable for comprehensive ankle therapy. Two typical examples of such robot platforms were developed by the rehabilitation robotics group at the University of Auckland [8, 16]. The parallel ankle robot by Tsoi et al. [16] is presented in Figure 3, where Figure 3(a) has six DOFs and Figure 3(b) has three rotational DOFs if a human user is using it and his/her shank is attached to the leg holder. While this ankle robot has shown potentials for clinical application, one limitation is that this device can only sit in an upright position due to the use of spherical joints (denoted as D in Figure 3) between the actuators and the fixed platform. More specifically, in the rightmost of Figure 3, the ankle robot can fall down in the direction of the arrow when sitting on a tilted position. An ideal robot structure for ankle rehabilitation should be continuously adjustable for patients with varying disabilities. This robot suits well with a patient in a sitting position but not if she/he wants a different position, as shown in Figure 3(c). To address this issue, some physical rotation axes can be considered to limit the motion of the end effector. This robot design for ankle rehabilitation has been demonstrated by Jamwal et al. [8] and Zhang [21], as in Figure 4. On one hand, this design can ensure the training safety of the patient by allowing compatible robot motion (only three rotations) with the ankle joint in Figure 4(a). One the other hand, the whole structure of the robot can be adjusted based on the actual needs of patients, as shown in Figure 4(b).

**Figure 5:**
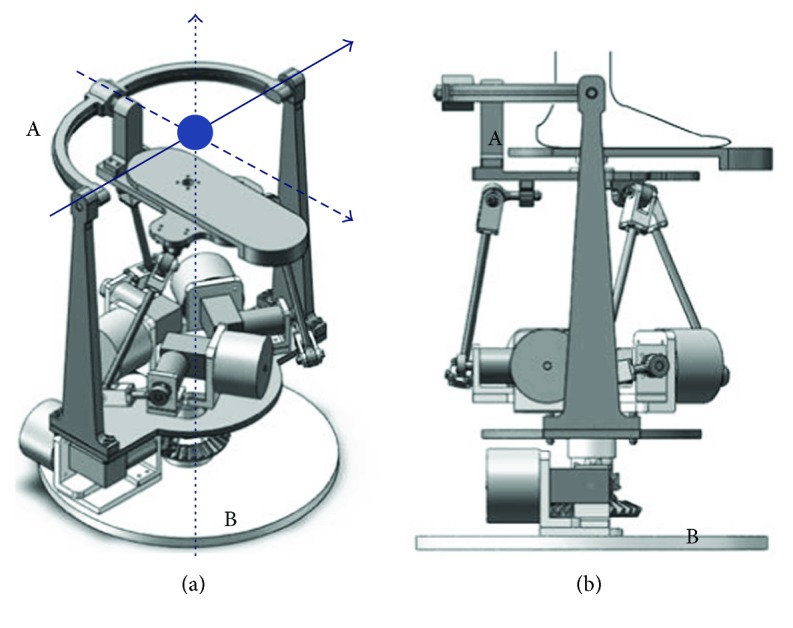
Different views of a parallel ankle robot developed by Wang et al. [22]: (A) the moving platform is essentially a two-link serial mechanism with two rotational DOFs and (B) the fixed platform. The blue dot represents the ankle joint; the blue solid line is the rotation axis of ankle dorsiflexion and plantarflexion; the blue dashdot line is the rotation axis of ankle inversion and eversion; the blue dot line is the rotation axis of ankle adduction and abduction actuated by a separate rotating motor.

While the robot developed by Jamwal et al. [8] and Zhang [21] shows great potential for ankle rehabilitation due to the use of a parallel mechanism with AaEE, the other way to achieve three DOFs and aligned rotation center has been proposed by Wang et al. [22], as shown in Figure 5. This novel 3-RUS/RRR redundant parallel ankle rehabilitation robot has their actuators below the end effector attached to a serial platform. Another advantage of this design is its small depth, which allows patients to easily put on no matter what size his/her leg is. It should be also noted that this robot cannot be adjusted to an arbitrary angle to fit the patient's sitting posture. Taking all into consideration, a robot design with three rotational DOFs, aligned rotation center, and adjustable posture structure can be considered to be suitable for comprehensive ankle training. A list of typical ankle rehabilitation robot designs is provided in Table 1.

To ensure comprehensive ankle exercises in a three-dimensional space, the requirement of three rotational DOFs can be a prerequisite of an optimal ankle rehabilitation robot. However, an ankle device with two DOFs for ankle (dorsiflexion/plantarflexion and inversion/eversion) is also acceptable since ankle adduction/abduction is limited and primarily controlled by rotation of the leg [27]. Therefore, as Wang et al. [22] suggested, an optimal ankle rehabilitation robot can be designed with the flexibility to be reconfigurable between two and three DOFs depending on the required training modes. In this way, the ankle robot by Zhang [21] can be further optimized by adding an electrical motor for the third revolute pair of the three-link serial mechanism. This robot can be operated with either two DOFs (dorsiflexion/plantarflexion and inversion/eversion) when the motor is locked or three DOFs in a synergic control. This approach can improve not only the singularity of the parallel mechanism, but also the achievable range of motion and actuation torque for ankle adduction and abduction.

With regard to the torque capacity of different ankle robots, many factors affect their performance, including the device structure and the selection of actuators. We focus on comparing and discussing these ankle devices with consistent rotation centers in terms of actuation capacity. The torque capacity of the motor-driven devices directly depends on the driving ability of the motor and the friction of the robot joint. A typical motor-driven ankle training device is the one developed by Zhang et al. [7]. Its output torque can be more than 10 Nm at extreme ankle positions, which meets the requirement of most ankle rehabilitation exercises, including joint stretching. It should be noted that resistive ankle exercises can require greater torque. This means a bulky workstation for the motor and reducer alongside the end effector/footplate. In using a serial mechanism for three-DOF ankle therapy, the robotic system will be more bulky and complex. By contrast, adopting parallel mechanisms for three-dimensional ankle exercises can make the robotic system more compact, and two devices, for example, are developed by Tsoi et al. [16] and Jamwal et al. [8], respectively. However, an issue of the parallel mechanism for ankle therapy is the conflict between robot workspace and actuation capacity. To achieve a compact robotic system, it is essential to select actuators with long stroke and high power-size ratio. In general, an appropriate actuation torque of an ankle rehabilitation robot depends on the defined robot function.

In terms of robot functionality, the installation of a variety of sensing components also affects the robot design. Wearable devices are more difficult to be integrated with a sensing system due to the operation in a gait pattern [5]. If going for a serial mechanism-based robot system with multi-DOFs, the whole structure can be heavy and bulky since an integrated actuation and sensing system is required along each link. For parallel mechanism-based robot systems without physical axes of rotations [16–18], difficulties exist in detecting the posture of the end effector unless using an optical tracking system or a gyroscope that mostly requires further data analysis. On a parallel mechanism with physical rotational axes, the integration of sensing components can be easily implemented with the robot structure, as discussed in [28].

In addition to the selection of robot structure, optimization techniques should be also applied to the robot to have a suitable workspace and improve its torque generation capacity. Dimensional synthesis is one of the most difficult issues in the field of parallel robots with or without actuation redundancy. To deal with the optimal design of a redundantly actuated parallel robot used for ankle rehabilitation, Wang et al. [19] presented a methodology of dimensional synthesis based on multiobjective optimization using a modified differential evolution algorithm. The objective functions separately reflect occupied space, input/output transmission, torque performances, and multicriteria constraints, such as dimension and interference. The design method proposed by Jamwal et al. [29] together with multiobjective optimization and fuzzy-based ranking can be generalized with modest efforts for the development of all of the classes of parallel robots. These techniques can provide guidance in designing and optimizing an ankle rehabilitation robot to ensure its excellent kinematics and kinetics performance.

### 4.2. Optimal Robotic Design for Ankle Rehabilitation

Robot-assisted ankle rehabilitation exercises can be delivered in a variety of ways to reduce motor impairment and enhance functional motor outcomes. While existing robot-assisted ankle rehabilitation techniques have shown great potential for treating ankle disabilities [4], few can deliver comprehensive ankle training in a three-dimensional space in passive and active modes with appropriate workspace and torque capacity. Depending on the patient's ankle disbility, rehabilitation robots can provide passive, active-assistive, active, and active-resistive exercises [1]. The predefined training trajecotries also vary for different levels of ankle injury. For example, stretching exercises for the treatment of drop foot are normally delivered along ankle dorsiflexion where the patients usually have difficulties in lifting their toes properly when walking [6, 12]. For treating ankle sprains, the predefiend trajectory can be anywhere in a three-dimensional space depending on injured muscles and ligaments [30].

By comparing a variety of ankle rehabilitation robots, it was found that an optimal robotic design for comprehensive ankle rehabilitation should be characterized with appropriate workspace and actuation torque, aligned rotation center between the robot and the ankle joint, and adjustable robot posture. However, this will definitely increase the cost due to complex robotic design. The authors also believe that no “one-size-fits-all” design exists for optimal robot-assisted ankle rehabilitation techniques, and therapy should be essentially tailored to each patient's needs and disabilities. Some examples of optimal ankle rehabilitation robots for certain applications are summarized in Table 2. The ankle stretching device developed by Zhang et al. [7] has been adaptively and intelligently controlled for various training exercises [31, 32] and can be well used for treating drop foot. The commercial product OptiFlex Ankle CPM is suitable for passive training with two DOFs. For comprehensive ankle exercises in a three-dimensional space, the compliant ankle rehabilitation robot developed by Zhang [21] has shown great potential for clinical applications. These three robotic prototypes are all characterized by an aligned rotation center between the robot and the ankle joint, appropriate robot workspace and actuation torque, and the ability of posture adjustability.

Additionally, Siegler et al. [26] have demonstrated that neither the ankle joint nor the subtalar joint acts as an ideal hinge joint of the ankle complex with fixed axes of rotations, and any foot motion is the result of rotations about both joints. Out of this consideration, further improvement can be made on existing ankle rehabilitation robots in terms of structural design. Taking the prototypes in Table 2, for example, the stretching device by Zhang et al. [7] has a fixed axis of rotation for ankle dorsiflexion and plantarflexion. To allow for a more biological design, the orientation of the rotation axis of the ankle stretching device can be redesigned to align with anatomical ankle joint and also with some floating flexibilities for training comfort and safety. The OptiFlex Ankle CPM has two fixed biological rotational axes, and thus, some floating design should also be brought to allow for moving rotational axes for training comfort and safety. To further improve the compliant ankle rehabilitation robot developed by Zhang [21], optimization should be conducted to make it achieve appropriate workspace and actuation torque. Further, as Qian and Bi [33] suggested, main obstacles of the promotion of rehabilitation robots in real life are due to the lacking of personalization and excellent cost performance. The use of a reconfigurable and modular architecture can be a good method to make a trade-off for this conflict. The ankle robot developed by Yoon and Ryu [34] can be used for ROM and muscle strengthening exercises, as well as the balance and proprioception training by adding an extra large plate. Similarly, the ankle robot developed by Zhang [21] can be also designed with configurability in generating varying workspace and actuation torque for subject/mode-specific training with enhanced safety.

However, two more design issues should be considered towards optimal ankle rehabilitation robots. One is the fixation issue of the human foot and shank, and a commonly used method is using straps attaching the foot on a footplate and the shank on a fixed leg holder. Again, since the anatomical ankle joint is not an ideal hinge joint [26], the rigid fixation of the foot and shank may result in discomfort and even injury risk. A potential solution can be to design the leg holder with some flexibilities to accommodate the difference between the robot structure and the human anatomy. The other issue is the setup of mechanical stops for training safety. Specifically, mechanical stops must be set on robotic systems whose workspace is larger than the actual ankle motions, especially on motor-driven devices. By contrast, these parallel ankle rehabilitation robots do not have to set mechanical stops if they are designed with appropriate workspace.

### 4.3. Limitations of This Review

An attempt was made to ensure that a variety of robotic designs proposed for ankle rehabilitation were reviewed. In this review, we assumed that the same robotic prototype reported in different studies and dates by the same group had the same design, although some minor improvements (such as using better materials or advanced control strategies) could have been made. However, other research may exist in which the ankle was not identified as a key term within the article; instead, some studies described the ankle complex as lower extremity or lower limb. Only articles after 1980 were included in this study as robot-assisted ankle rehabilitation techniques were quite limited before then. We included published journal and conference papers with a clear description of the robot design but did not include those written in languages other than English. Some studies may therefore have been excluded on this basis, leading to a potentially incomplete search.

## 5. Conclusions

This review focuses on the design analysis of existing ankle rehabilitation robots. Although most robot-assisted ankle rehabilitation techniques have been demonstrated to be effective for ankle physical therapy, they have drawbacks in the design that have impeded their applications in a wide range. Comparative analysis indicates the following:

1. An optimal ankle rehabilitation robot design must be characterized with aligned rotation center as the ankle joint, appropriate workspace, and actuation torque.
2. The number of robot DOFs depends on specific applications. The single-DOF robot is mostly developed for a special application such as ankle stretching along dorsiflexion and plantarflexion, while multi-DOF devices are more suitable for comprehensive ankle rehabilitation exercises.
3. Other factors, including the adjustability of robot posture, sensing functions, the fixation of the human foot and shank on the robot, and mechanical stops, also affect their clinical applications. Adjustable robot posture can enable its use on a large population of patients with varying ankle disabilities. Robots with all required sensing functions allow for the implementation of advanced interactive training. The leg holder should be designed with some flexibilities to accommodate the difference between the robot structure and the human anatomy. Mechanical stops must be set for training safety especially on robotic systems whose workspace is larger than the actual ankle motions.
4. Ankle rehabilitation robots with reconfigurability will be a new research area towards optimal design with appropriate workspace and actuation torque, especially on parallel mechanisms. Multiobjective optimization techniques can be involved to make robots in optimal kinematic and dynamic performance.

## Figures and Tables

**Table 1:** Characteristics description of ten typical ankle rehabilitation robots.

Ankle robot	DOF	Type	Alignment	Posture adjustability	Supplementary information
Yu et al. [15]	1	Wearable	Yes	No	This robot system consists of an ankle-foot module and a knee module, specially designed for gait training.
Park et al. [5]	2	Wearable	Yes	Yes	The prototype generates an ankle range of motion (ROM) of 27° (14° dorsiflexion and 13° plantarflexion). This is acceptable for gait training rather than ankle stretching due to limited workspace.
Roy et al. [6]	2 (3)	Wearable	Yes	Yes	The MIT Anklebot allows normal ROM in all three DOFs of the foot during walking overground, on a treadmill, or while sitting. Two DOFs are actively actuated by motors.
Zhang et al. [7]	1	Platform	Yes	Yes	This device is portable and low cost, making it available to patients for frequent and long-term use at clinics or home.
OptiFlex Ankle CPM [20]	2	Platform	Yes	Yes	A commercial device for full ankle ROM exercises along dorsiflexion/plantarflexion and inversion/eversion.
Girone et al. [18]	6	Platform	Depending on control design	Yes	Limited robot workspace if controlled for aligned rotation center between the robot and the ankle joint.
Saglia et al. [3]	2	Platform	No	Yes	This robot used customized linear actuator to meet the required forces and torques for strengthening and balance exercises.
Tsoi et al. [16]	3 (6)	Platform	Yes	No	This robot itself has six DOFs and three rotational DOFs if considering the human ankle as a constraint. It can be only used in an upright posture.
Jamwal et al. [8]	3	Platform	Yes	Yes	Limited actuation torque for patient-robot interactive training.
Zhang [21]	3	Platform	Yes	Yes	Optimization techniques should be involved to enhance the usability and functionality of this robot.
Wang et al. [22]	2 or 3	Platform	Yes	Yes	This device has the flexibility of reconfiguring into either a 2-DOF rehabilitation device or a 3-DOF one depending on the specific exercise mode. But it has not been validated experimentally.

Note: the robot degrees of freedom are calculated based on the famous Chebyshev-Grubler-Kutzbach criterion [23]. Degrees of freedom of a mechanism *F* = *λ*(*n* − *j* − 1) + ∑_*i*=1_^*j*^*f*_*i*_ − *f*_p_, where *λ* refers to the degrees of freedom of the space, *n* is the number of links including the base, *j* is the number of binary joints, *f*_*i*_ is the degrees of relative motion permitted by joint *i*, and *f*_p_ is denoted for the total number of passive degrees of freedom.

**Table 2:** Examples of ankle rehabilitation robots with optimal design for different applications.

Examples	DOF	Type	Alignment^∗^	Posture adjustability^∗^	Workspace^∗^	Actuation torque^∗^	Application fields
Zhang et al. [7]	1	SM-based platform	Yes	Yes	Appropriate	Appropriate	+
OptiFlex Ankle CPM [20]	2	SM-based platform	Yes	Yes	Appropriate	Appropriate	++
Zhang [21]	3	PM-based platform	Yes	Yes	Optimization should be conducted for appropriate robot workspace and actuation torque, and reconfigurable design should be also encouraged.		+++

SM: serial mechanism; PM: parallel mechanism.

Note: ∗ represents the characteristics an optimal ankle rehabilitation robot must have; + refers to the fields of intelligent ankle stretching only for dorsiflexion/plantarflexion; ++ refers to the fields of passive training for ankle inversion/eversion and adduction/abduction; +++ refers to the fields of comprehensive ankle training in a three-dimensional space.
